# A42 HETEROGENEITY IN EFFICACY AND SAFETY ENDPOINTS FOR PEDIATRIC CLINICAL TRIALS IN INFLAMMATORY BOWEL DISEASE: A NEED FOR HARMONIZATION

**DOI:** 10.1093/jcag/gwab049.041

**Published:** 2022-02-21

**Authors:** E Crowley, D Turner, C Ma, T Nguyen, H McKay, R Schneider, A Silverberg, A Muise, B Feagan, A Griffiths, V Jairath

**Affiliations:** 1 Alimentiv Inc, London, ON, Canada; 2 University of Calgary, Calgary, AB, Canada; 3 Shaare Zedek Medical Center, Jerusalem, Jerusalem, Israel; 4 The Hospital for Sick Children, Toronto, ON, Canada; 5 Western University, London, ON, Canada

## Abstract

NOT PUBLISHED AT AUTHOR’S REQUEST

**Funding Agencies**: None

